# Metagenomic insights into the effects of cosmetics containing complex polysaccharides on the composition of skin microbiota in females

**DOI:** 10.3389/fcimb.2023.1210724

**Published:** 2023-08-01

**Authors:** Shumao Cui, Mingluo Pan, Xin Tang, Guangrong Liu, Bingyong Mao, Jianxin Zhao, Kaiye Yang

**Affiliations:** ^1^ State Key Laboratory of Food Science and Resources, Jiangnan University, Wuxi, China; ^2^ School of Food Science and Technology, Jiangnan University, Wuxi, China; ^3^ Infinitus R&D Center, Infinitus (China) Company Ltd, Guangzhou, China

**Keywords:** 16S rDNA, skin bacterial microbiota, cosmetics containing complex polysaccharides, forehead, cheek

## Abstract

**Introduction:**

The use of cosmetics has become a habit for women. However, their influence on the microbial diversity of the skin has rarely been studied.

**Methods:**

Herein, the effect of cosmetics containing complex polysaccharides on the skin bacterial microbiota of female forehead and cheek areas was analyzed. Eighty volunteers were recruited and split into two groups (40 people each); one group was treated with cosmetics containing complex polysaccharides and the other with basic cream for 28 days. Skin samples were collected using sterilized cotton swabs, and 16S rDNA high-throughput sequencing was used to analyze the changes in skin bacterial microbiota composition before and after the intervention.

**Results and discussion:**

A total of twenty-four phyla were detected in the forehead and cheek skin samples of 80 volunteers, the top three of which were Proteobacteria, Firmicutes, and Actinobacteria. The main genera of the forehead skin bacterial microbiota were *Cutibacterium* (11.1%), *Acinetobacter* (10.4%), *Enterococcus* (8.9%), *Ralstonia* (8.8%), and *Staphylococcus* (8.7%), while those of the cheek skin bacterial microbiota were *Staphylococcus* (20.0%), *Ralstonia* (8.7%), *Propionibacterium* (7.9%), *Acinetobacter* (7.2%), and *Bifidobacterium* (6.0%). Compared with basic cream, the use of cosmetics containing complex polysaccharides significantly increased the relative abundance of Staphylococcus and Bacillus in the forehead and cheek and reduced the relative abundance of *Propionibacterium* and *Bifidobacterium*. Thus, cosmetics containing complex polysaccharides could modify the composition of skin bacterial microbiota, which may help to maintain stable conditions of the skin.

## Introduction

The skin is the largest organ of the human body. It is often exposed to a variety of external environments, forming a barrier to ensure the dynamic balance of the entire body against harmful environmental effects (Nemes and Steinert, 1999). As the first line of defense against pathogens, the skin is home to several microbes, which can effectively improve the host’s immune function (Manus et al., 2020). Similar to the gut microbiota, the surface microbiota of skin remains relatively stable over time in the absence of major disturbances, despite exposure to the external environment (Faith et al., 2013; Oh et al., 2016). In healthy people, the skin itself has the ability to maintain the skin microbial structure, which can be a marker of skin health. By contraries, microbiota changes can often be observed in several skin diseases, such as acne (Gallo and Nakatsuji, 2011), atopic dermatitis (Zhang et al., 2011), eczema (Chularojanamontri et al., 2016), and psoriasis (Alekseyenko et al., 2013).

Over the past few decades, considerable research on the microbes that live on human skin has been done. In general, skin microbes can be divided into residential and transient bacteria (Swaney and Kalan, 2021). Resident bacteria are a type of skin bacteria that grow on the surface of the epidermis or the stratum corneum with a relatively fixed number and type. Common resident bacteria include *Micrococcus*, *Corynebacterium*, and *Propionibacterium*. There are also some bacteria that come from the contact between people and the external environment, which temporarily exist on the surface of the skin, and whose number and types often vary greatly. We refer to these as temporary bacteria or passerby bacteria (Wilson, 2005; Staudinger et al., 2011). These bacteria include *Staphylococcus aureus* and *Streptococcus hemolyticus*. Different skin parts of the human body have distinct characteristics, which leads to significant differences in the composition of the bacterial microbiota. Based on the differences in physiological characteristics, the skin can be divided into dry areas (arms, legs, buttocks, etc.), wet areas (armpits, belly button, groin, etc.), and oily areas (forehead, cheeks, chest, back, etc.) (Cundell, 2018). The dry area is mainly composed of Proteus, Bacteroides, and Actinomycetes (James, 2020). The wet zone provides a moist niche for many microorganisms, causing the number of bacteria in this area to be significantly higher than in other areas. The most common bacteria in this area are *Staphylococcus* and *Corynebacterium (* Marples, 1965; Flowers and Grice, 2020). The oily zone is an area of the skin with many sebaceous glands whose secreted sebum is a source of lipids required for the growth of many microorganisms. Therefore, it is the best environmental area for the growth of many lipophilic microorganisms, with Propionibacterium and Staphylococcus being predominant (Capone et al., 2011; Grice and Segre, 2011; Malinowska et al., 2017). As the forehead is more prone to secrete oily substances, the sebum level is higher than in other facial areas such as the cheeks; thus, the microbial composition of the forehead may also be significantly different from other oily areas (Lopez et al., 2000; Tagami, 2008; Lee et al., 2013).

Skin conditions is closely related to skin microorganisms, and the two complement may regulate each other. For example, the skin can release neurohormones, such as peptides and catecholamines, to modify the activity of skin microorganisms. Meanwhile, a study has shown that skin microorganisms can release neurohormonal compounds such as histamine, glutamic acid, and r-aminobutyric acid to improve the physiological conditions of the skin (Cundell, 2018). The study of intestinal bacterial microbiota regulating intestinal health reminds us that we can improve the balance of the skin by regulating the skin bacterial microbiota. Cosmetics, especially skin care products, are used to improve the quality of the skin and delay skin aging. These cosmetics modify the diversification of skin microorganisms by adding some active ingredients that are beneficial to or inhibit the growth of certain microorganisms and achieve skin care effects after long-term or regular use (Paluszak et al., 2011). Studies have shown that mycosporin-like amino acids (MAAs) are used as active ingredients in cosmetics to achieve sun protection because they can dissipate ultraviolet energy without generating free oxygen radicals (Kageyama and Waditee-Sirisattha, 2019). The metabolites of *Streptomyces* have excellent antioxidant and anti-inflammatory properties, which can be used as potential cosmetic additive ingredients (Sánchez-Suárez et al., 2020). Polysaccharides are an important part of the human skin, and the addition of anticoagulant polysaccharides in cosmetics can improve skin’s metabolism. Robert et al. have shown that fucose polysaccharides can slow down human skin ageing and even can reverse age-dependent skin alterations (Robert et al., 2003). In addition, another study found that fucoidan significantly inhibited the adhesion of *Staphylococcus aureus* and *Cutibacterium acnes*, which can help in the treatment of atopic dermatitis (Park et al., 2021). Furthermore, Péterszegi et al. also found that the addition of fucoidan *in vitro* cell culture can promote the proliferation of fibroblasts (Péterszegi et al., 2003). These indicates that polysaccharides can repair skin tissues by activating cell growth factors. However, the effect of polysaccharide cosmetics on the skin bacterial microbiota has rarely been studied. Therefore, in this study, we explored the effect of cosmetics supplemented with complex polysaccharides on the microbiota of women’s forehead and cheek skin.

## Materials and methods

### Subject selection and sampling

During the development of this project, a total of 80 female volunteers with no history of skin diseases were recruited, of which 74 volunteers completed the entire trial (Among them, 6 subjects withdrew from the trial due to personal reasons). Volunteers were between 20 and 55 years of age, with an average age of 30 years. The volunteers were randomly divided into two groups (Group A and B), each with 37 participants. Group A used basic essence water (containing propylene glycol and deionized water, pH 6-7) as the control group, while group B used essence water containing polysaccharides as the experimental group. The polysaccharides were mixtures of three kinds of polysaccharides extracted from tremella, dendrobium, and snow lotus, and the ratio of the three polysaccharides were 1:1:1, respectively. *The polysaccharides extracted from tremella and dendrobium were obtained from Nutri-Woods Bio-tech (Beijing) Co., Ltd (Beijing, China). The polysaccharides extracted from snow lotus were obtained from Sanyou Bio-tech (Beijing) Co., Ltd. (Beijing, China).* The cosmetics were applied in the morning and evening, with the entire intervention period lasting for 4 weeks. We collected the volunteers*’* forehead and cheek swab samples on days 0, 14, and 28. The subjects avoided washing their face or applying topical medications and emollients 24* h* before the sample was collected. According to the description in the Human Microbiome Project (Aagaard et al., 2013), a sterile cotton swab of Tris-EDTA and 0.5% Tween20 solution was used to repeatedly wipe an area of about 4×4 cm above the sampling site with the appropriate pressure for approximately 30 s. The cotton swab head was then cut with sterile medical scissors and placed in a sterile 2 mL-centrifuge tube. All samples were stored at -80°C before further processing.

This study was approved by the Ethics Committee in Jiangnan University, China (SYXK 2012-0002). All the skin samples from volunteers were for public health purposes and these were the only human materials used in present study. Written informed consent for the use of their skin samples were obtained from the participants. All of them conducted health questionnaires before sampling and no human experiments were involved.

### DNA extraction and quality control

The FastDNA Spin Kit for soil (MP Biomedical, Irvine, CA, USA) was used to extract the genomic DNA of the sample, according to the manufacturer**’**s instructions. The extracted DNA was dissolved in sterile water and the quality was evaluated by NanoDrop 2000 Spectrophotometer (Thermo Fisher Scientific, United States). Finally, all high-quality genomic DNA were stored in a refrigerator at -20**°**C.

### 16S rRNA gene amplification, sequencing, and bioinformatic analyses

The extracted genomic DNA was used as a template, while the primers (341F: CCTAYGGGRBGCASCAG, 806R: GGACTACNNGGGTATCTAAT) were used to amplify the V3-V4 region of the 16S rDNA, with the fragment size being 466 bp. Different samples were distinguished by a barcode (5 **‘**-terminal) consisting of seven bases. The reaction system consists of (50 μL): 2 μL of template, 0.5 μL of 341F upstream primer, 0.5 μL of 806R downstream primer, 25 μL of 2×Taq PCR master mix, and 22 μL of enzyme-free water. The PCR program was: pre-denaturation at 95**°**C for 5 min, followed by 30 cycles of denaturation at 95°C for 30 s, annealing at 55°C for 30 s, extension at 72°C for 1 min, and a final extension at 72°C for 7 min. The PCR products were analyzed by 2% agarose gel electrophoresis, and the target fragments were cut out and purified using a gel recovery kit (Tiangen, Beijing, China). The purified samples were quantified using Qubit 3.0 (Life Technologies, CA, USA), mixed with equal quality to prepare a gene library, and then used in the PE 300 kit (Illumina, San Diego, CA, USA) on the Illumina MiSeq platform according to the manufacturer**’**s instructions.

The raw data were demultiplexed and quality filtered using the q2-demux plugin followed by denoising with DADA2 (Callahan et al., 2016) plug-in in QIIME2 (Bolyen et al., 2019). All amplicon sequence variants (ASVs) were classified by the q2-feature-classifier. For analyzing the microbiota alpha and beta diversity, all reads was randomly rarefied to 10,000 reads per sample.Linear discriminant analysis (LDA) effect size (LEfSe)**)** (Segata et al., 2011)was used to analyze the microbial community differences between different groups (LDA>3.0, p<0.05), using the online analysis website http://huttenhower.sph.harvard.edu/galaxy/.

### Statistical analysis

Skin bacterial microbiota compositional differences between the different groups were analyzed using GraphPad Prism 8 (GraphPad Software Inc., San Diego, CA, USA) for the Kruskal-Wallis non-parametric test. α diversity (observed OTUs and Shannon) differences were analyzed by one-way analysis of variance (ANOVA), followed by Dunn**’**s multiple comparison tests. Its difference with β diversity is that it uses two algorithms, weighted UniFrac and Bray–Curtis distances, and performs a PERMANOVA test. All graphics rendering used R 3.6.1. Statistical significance was set at *P* < 0.05.

## Results

### Characteristics of the forehead and cheek bacterial microbiota of females

**Figure 1:**
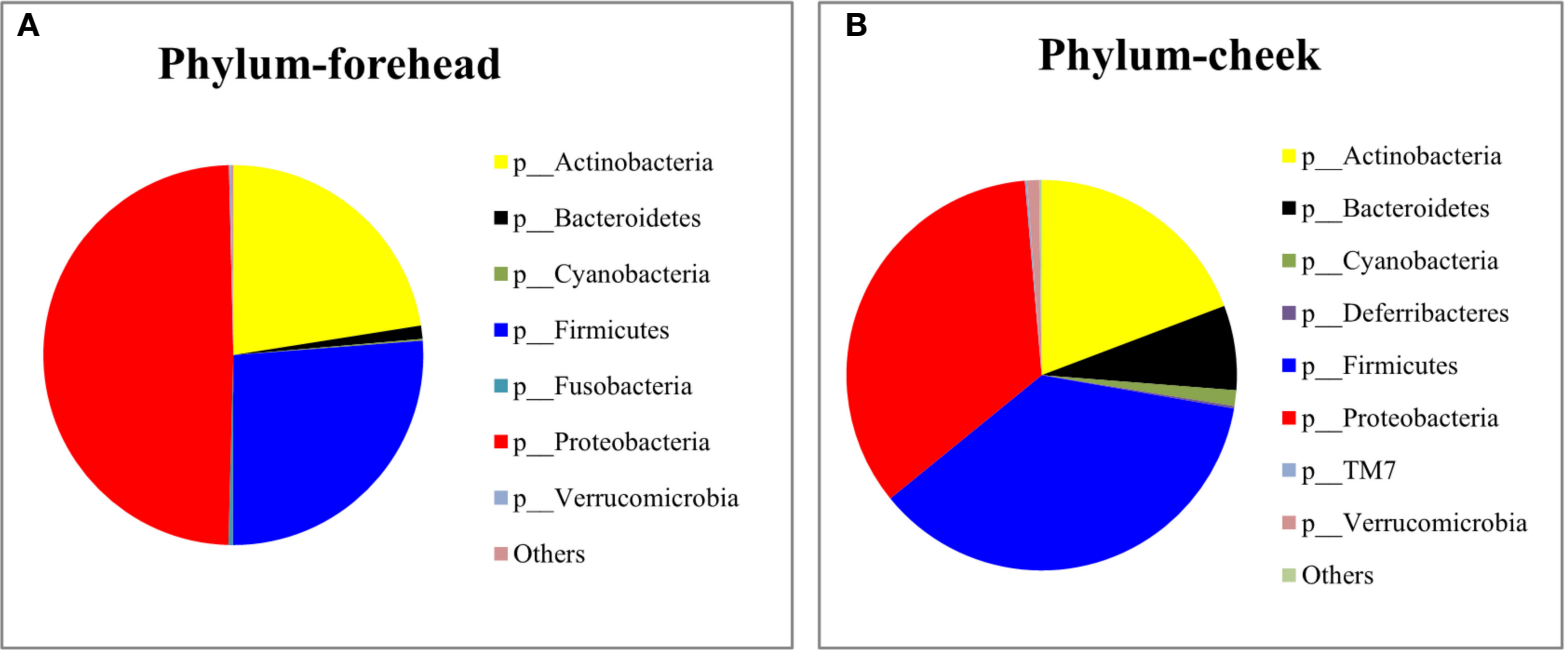
Relative abundance of the most predominant phylum on the forehead **(A)** and cheek **(B)**.

The baseline skin bacterial microbiota of the 80 volunteers*’* forehead and cheek skin samples were sequenced for the 16S rDNA V3-V4 region. After sequence splicing, quality filtering, and chimera removal, 3,157,972 valid sequences were retained for subsequent downstream analysis. Based on the nucleotide sequence homology of =100%, the 16S rDNA gene sequence was clustered into 1,023 features. The Silva-138-99 database of 16S rDNA was used for comparison. The annotation results revealed that a total of 24 phyla were detected in the forehead and cheek skin samples of the 80 volunteers. The top three phyla of the forehead skin bacterial microbiota were Proteobacteria (49.22%), Firmicutes (26.26%), and Actinobacteria (22.51%), while for the cheek skin bacterial microbiota, the top three phyla were Firmicutes (36.4%), Proteobacteria (34.5%), and Actinobacteria (19.7%). The cheek skin Firmicutes were significantly higher than that of the forehead, while the Proteobacteria were significantly lower. In addition, small amounts of Bacteroidetes, Cyanobacteria, Fusobacteria, Deferribacteres, and TM7 were detected in the skin of the cheeks and forehead (**Figures 1A, B**). Among all forehead and cheek skin samples, 18 and 27 (including 8 unclassified) genera, respectively, were detected with a percentage of more than 1%. Cutibacterium (11.1%), Acinetobacter (10.4%), Enterococcus (8.9%), Ralstonia (8.8%), and Staphylococcus (8.7%) accounted for the highest proportion of the forehead skin bacterial microbiota, whereas the main genera in the cheek skin bacterial microbiota were Staphylococcus (20.0%), Ralstonia (8.7%), Propionibacterium (7.9%), Acinetobacter (7.2%), and Bifidobacterium (6.0%).

### Effect of cosmetics on the composition of forehead and cheek skin bacterial microbiota

#### Effect of basic cream on forehead and cheek skin bacterial microbiota (group A, 0 days VS 14-28 days)

##### Analysis of skin bacterial microbiota composition

**Figure 2:**
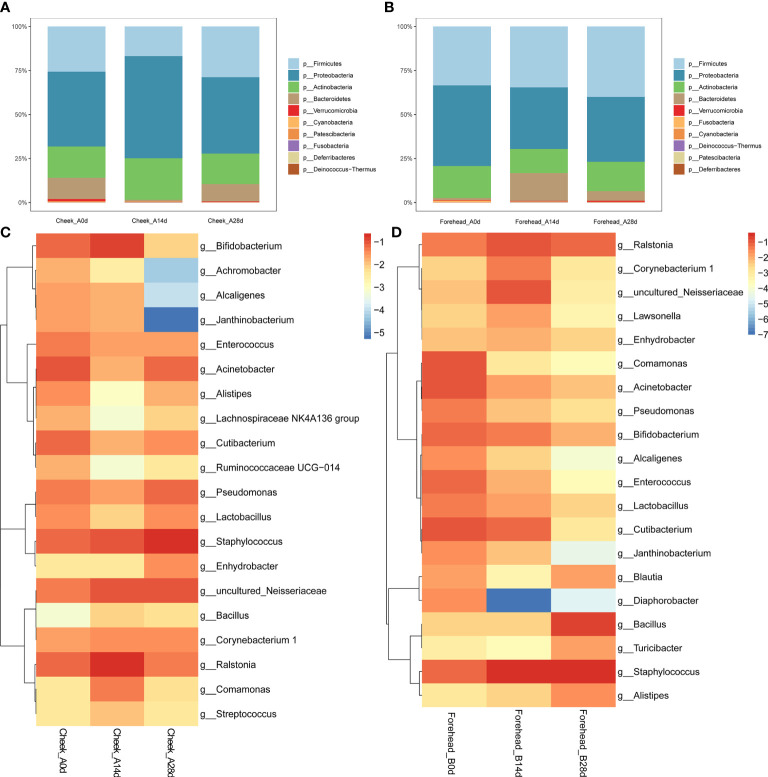
**(A, B)** Relative abundance of the phyla on the forehead **(A)** and cheek **(B)**. **(C, D)** Heatmap of the proportion of the 20 operational taxonomic units (OTUs) determined as dominant bacteria.

As shown in **Figure 2A**, after 28 days of continuous use of the basic cream, the forehead skin microbiota showed a certain degree of change. At the phylum level, during days 14 to 28, the relative abundance of Firmicutes gradually increased, but compared with day 0, the relative abundance of Firmicutes did not change significantly. In addition, the relative abundance of Proteobacteria and Bacteroidetes increased significantly. **Figure 2B** shows that the abundance of Proteobacteria in the cheek skin bacterial microbiota increased at day 14 (37.4% to 54.7%) but returned to a level similar to that at day 0 (43.3%) after 28 days. The relative proportion of Firmicutes decreased from 22.6% to 15.8% at day 14 and recovered to 28.7% at day 28.

At the genus level, the relative abundance of Staphylococcus, Bacillus, and Bifidobacterium in the bacterial microbiota of forehead skin increased after 28 days of use of the basic cream. However, the relative abundance of Ralstonia, Alistipes, and Blautia decreased. The results of the significance analysis showed that the changes in the above-mentioned genera did not reach a significant level (p<0.05). Similarly, no significant changes were found in the cheek skin bacterial microbiota (**Figures 2C, D**).

#### Effect of cosmetics containing complex polysaccharides on forehead and cheek skin bacterial microbiota (group B, 0 days VS 14-28 days)

##### Analysis of skin bacterial microbiota composition

**Figure 3:**
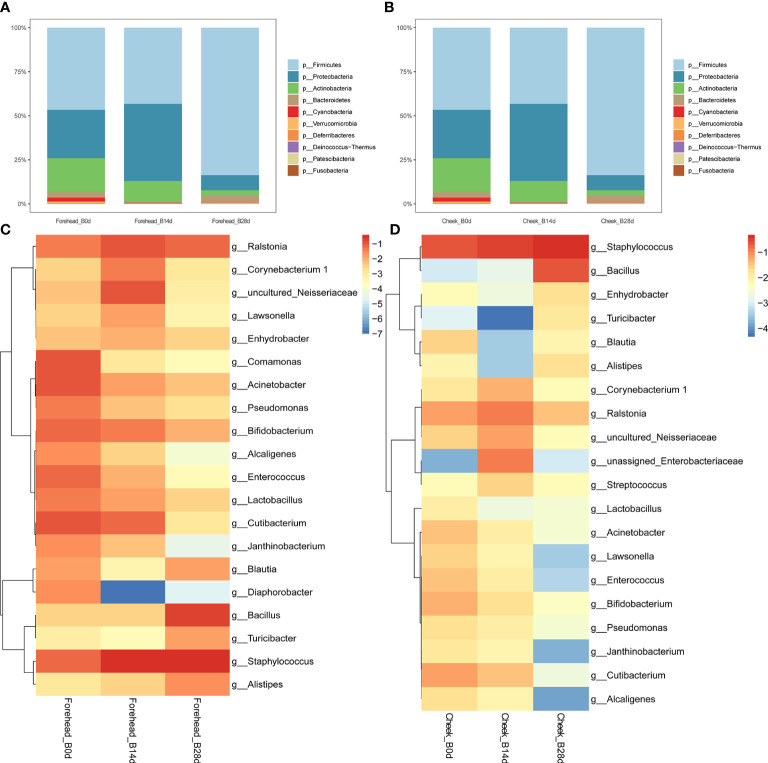
**(A, B)** Relative abundance of phyla on the forehead and cheek. **(C, D)** Heatmap of the proportion of the 20 operational taxonomic units (OTUs) determined as dominant bacteria.

**Figure 4:**
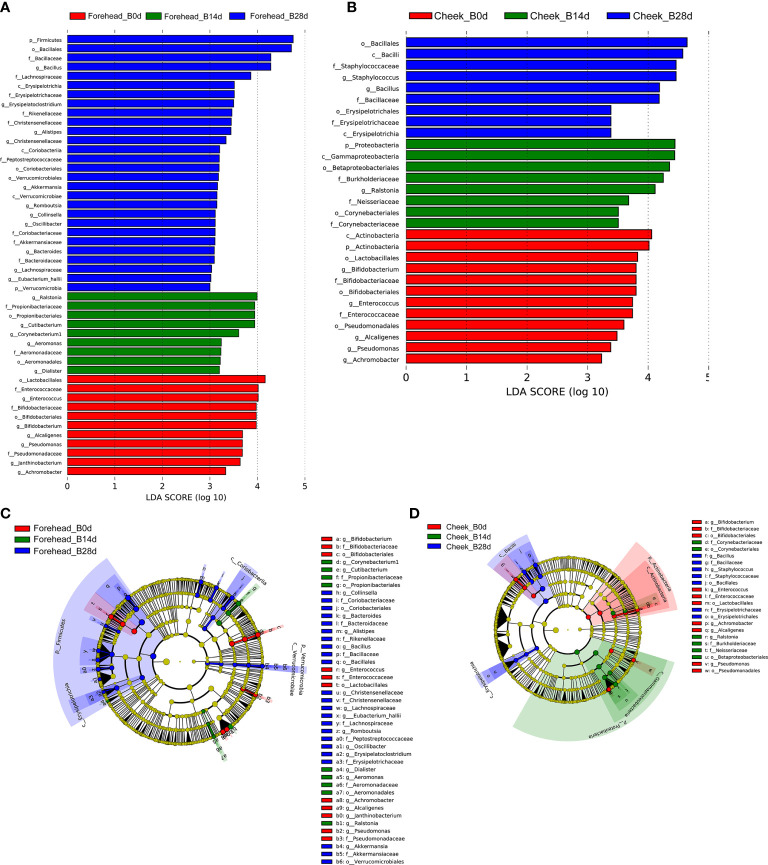
**(A, B)** LDA scores of skin microbiota for the different groups between forehead and cheek. **(C, D)** Cladograms represent the LEfSe results for the different groups between the forehead and cheek.

It can be seen from the **Figure 3** that after 28 days of continuous use of cosmetics containing complex polysaccharides, the composition of the forehead and cheek skin bacterial microbiota has undergone major changes. At the phylum level, with the use of complex polysaccharide cosmetics, the relative abundance of Firmicutes in forehead skin gradually increased from 20.9% to 82.1%. Combined with the LEfSe analysis, it was found that Firmicutes had an LDA score of (log10)>3 and a p value of *<*0.05 (**Figures 3A, C, D**) after 28 days, indicating that the relative abundance of Firmicutes increased significantly. In addition, the relative abundance of Bacteroidetes in the forehead skin bacterial microbiota also increased to a certain extent (0.9%–4.9%). Correspondingly, the percentages of Proteobacteria and Actinobacteria in the forehead bacterial microbiota gradually decreased from 39.0% to 8.7% and 19.4% to 2.8%, respectively. For the cheek skin bacterial microbiota, the relative abundance of Firmicutes decreased slightly at day 14, but it also increased significantly after 28 days. However, Proteobacteria showed the opposite trend. For Actinobacteria, it was gradually reduced from 15.7% of the baseline to 3.1% (**Figure 3B**), and LEfSe analysis showed that the LDA score was (log10)>3 and the p value was *<*0.05 (**Figures 4A, C**).

At the genus level, the relative abundance of Staphylococcus, Bacillus, Blautia, Alistipes, and Ralstonia in the forehead skin bacterial microbiota increased after 28 days of using the complex polysaccharide cosmetics (**Figure 3C**). Combined with the LEfSe analysis, we found that the relative abundance of Bacillus, Alistipes, Akkermansia, Bacteroides was significantly increased (LDA score of (log10)>3 and p *<*0.05) (**Figures 4A, C**). For the cheek skin bacterial microbiota, the relative abundance of Staphylococcus, Bacillus, and Alipipes was significantly increased, while the relative abundance of Pseudomonas and Bifidobacterium was significantly decreased (**Figure 3D**, **Figures 4B, D**).

##### α-diversity and β-diversity analysis

**Figure 5:**
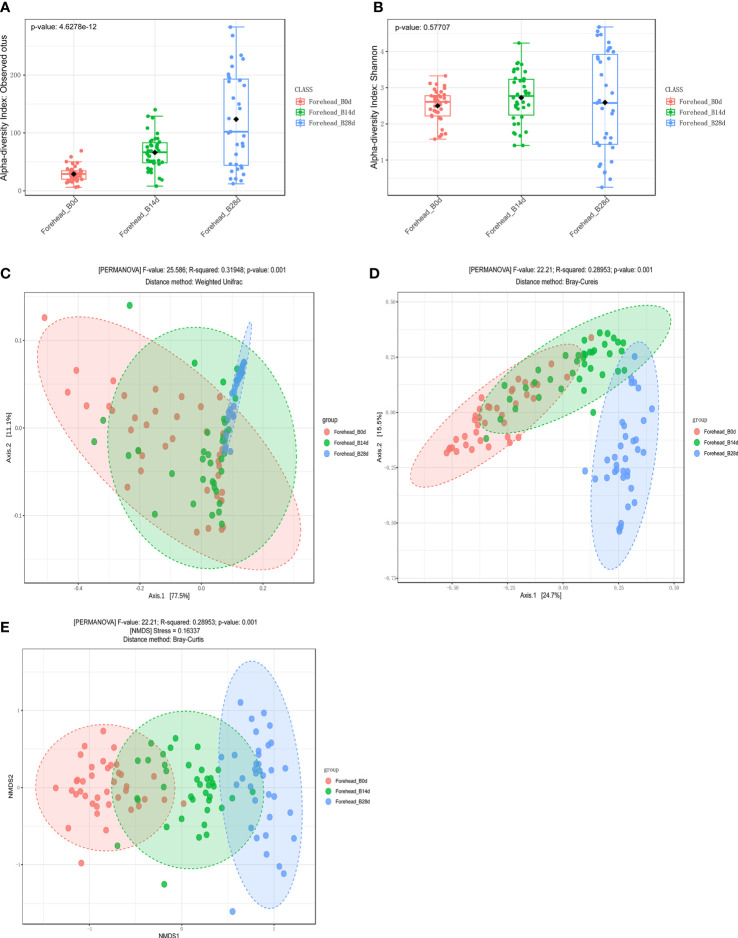
**(A, B)** *α diversity indices of forehead* skin microbiota for the different groups. **(C, D)** Principal coordinate analysis (PCoA) plots by Weighted UniFrac and Bray–Curtis distances (PERMANOVA, p < 0.001). **(E)** NMDS plots displaying samples by different periods using Bray–Curtis distance (PERMANOVA, p < 0.001).

**Figure 6:**
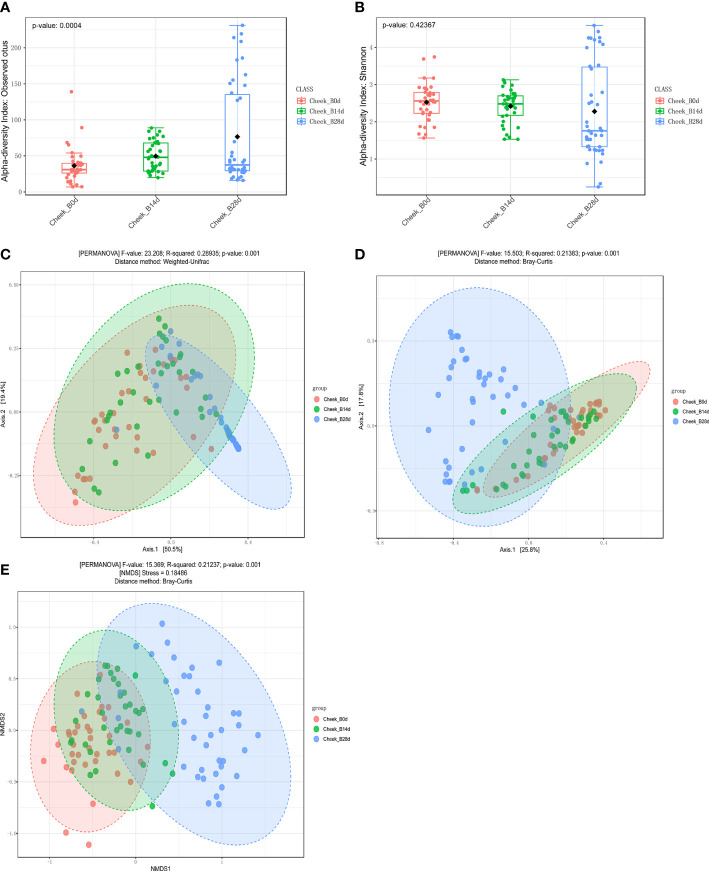
**(A, B)** α diversity indices of cheek skin microbiota for the different groups. **(C, D)** Principal coordinate analysis (PCoA) plots by Weighted UniFrac and Bray–Curtis distances (PERMANOVA, p < 0.001). **(E)** NMDS plots displaying samples by different periods using Bray–Curtis distance (PERMANOVA, p < 0.001).

Compared with day 0, *observed OTUs of forehead skin bacteria microbiota* significantly increased after 14 days of using *cosmetics containing complex polysaccharide*s, and further increased after 28 days (p<0.001) (**Figure 5A**). The Shannon index also gradually increased with time (**Figure 5B**), but did not reach a significant level (p=0.57). The principal coordinate analysis (PCoA) results of the two algorithms (weighted UniFrac and Bray–Curtis) showed that the samples of the same number of days of using the cosmetics containing complex polysaccharides can be clearly clustered and can be significantly distinguished from the 0-day samples (p<0.001) (**Figures 5C, D**). At the same time, using NMDS to cluster sample OTU levels yielded the same result (**Figure 5E**). The analysis results of α-diversity and β-diversity of cheek skin bacterial microbiota were similarly changed (**Figure 6**). Moreover, analysis of skin microbiota in group A found that the use of basic cream did not significantly change the composition and diversity of skin microbiota (the results are shown in the supporting materials). Therefore, the analysis of α-diversity and β-diversity shows that the use of complex polysaccharide cosmetics has a significant impact on the composition of the forehead and cheek skin bacterial microbiota.

## Discussion

In this study, we examined the effect of the addition of complex polysaccharides to cosmetics on the composition and diversity of forehead and cheek skin bacterial microbiota of females. In the absence of major external interference, the human skin bacterial microbiota maintains a relatively stable state. Numerous studies on the microbial composition of human skin have shown that the most abundant phyla on the forehead and cheeks are Actinobacteria, Firmicutes, and Proteobacteria (Dekio, 2005; Grice et al., 2009; Staudinger et al., 2011). Our research produced similar results; however, some differences were also observed. For the phylum Bacteroides, Staudinger (Staudinger et al., 2011) believed that it would occur only on the forehead of women, but we detected it on the forehead as well as cheeks of women, and the relative abundance on the cheeks was slightly higher than on the forehead (7.0% vs. 1.13%). Souvik identified approximately 1,000 genera in the forehead and cheek regions of 30 healthy women, including *Propionibacterium*, *Staphylococcus*, *Streptococcus*, *Corynebacterium*, and *Paracoccus* (Mukherjee et al., 2016). In addition to detecting bacteria similar to those by Souvik, we found that *Acinetobacter* (10.4%), *Enterococcus* (8.9%), and *Ralstonia* (8.8%) also accounted for a certain proportion of the forehead skin bacterial microbiota. *Ralstonia* (8.7%), *Acinetobacter* (7.2%), and *Bifidobacterium* (6.0%) were also found in the cheek skin bacterial microbiota. With the deepening of research on human skin microorganisms, more bacterial genera may be discovered.

Cosmetics are designed to produce specific effects on the skin, including cleansing, fragrance, changing appearance, correcting unpleasant odors, and maintaining good conditions. These effects may change the microbial community in the human skin. Researchers may add certain substances to achieve the desired effect when designing cosmetics. For example, the use of 0.5%–1.5% methyl parahydroxybenzoate in cosmetics has a significant inhibitory effect on *Propionibacterium acnes* and *Staphylococcus epidermidis*, in order to treat acne (Cai et al., 2013). In addition, there are some molecules that directly or indirectly provide nutrition for skin microorganisms, such as glycerin, amino acids, hydrolyzed collagen, and polysaccharides (Robert et al., 2003; Paluszak et al., 2011; Kageyama and Waditee-Sirisattha, 2019; Sánchez-Suárez et al., 2020; Pinto et al., 2021). The skin care properties of bioactive polysaccharides include anti-aging, vascular beauty, anti-acne, skin tissue repair, whitening, and moisturizing effects (Péterszegi et al., 2003; Robert et al., 2003). Whether adding polysaccharides to cosmetics will cause changes to the skin microbiota or what changes will be produced is the core question of this research. Regarding the issue of the use cycle of cosmetics, some studies have only been conducted for a brief period of 7–10 days, leading to an incomplete assessment of skin microbial changes, because the skin renewal process generally takes 21–28 days (Houben et al., 2006; Blanpain et al., 2007). Therefore, our research period was 28 days. The β diversity results also showed that the samples from 0–14 days were not clearly separated in the coordinate system, while the samples at 28 days were significantly separated from the other two time points. Our research also found that compared with the control group using the basic cream, the group using the cosmetics containing complex polysaccharides had the number and diversity of their skin bacterial microbiota increased to a certain extent. Previous research on intestinal bacterial microbiota indicates that a reduction in the number and diversity of microorganisms may disrupt the balance of the bacterial microbiota, leading to illnesses in the body. On the contrary, an increase in the diversity of the microbiota is considered a beneficial effect. There are also studies suggesting that inappropriate use of cosmetics can lead to negative effects on biological disorders by reducing the diversity of skin bacterial microbiota (Bouslimani et al., 2019). Therefore, we infer that complex polysaccharide cosmetics can help to maintain stable conditions by increasing the number and diversity of skin bacterial microbiota.

Specifically, the composition of the microbiota of the volunteers changed significantly after 28 days of using the cosmetics containing complex polysaccharides. For the analysis at the genus level, the increased abundance of Firmicutes after the use of cosmetics containing complex polysaccharides was mainly contributed by *Staphylococcus* and *Bacillus*. However, the decrease in the relative abundance of Actinobacteria was mainly due to a decrease in the relative abundance of *Propionibacterium* and *Bifidobacterium*. A study has shown that *Staphylococcus epidermidis* can mediate the fermentation of glycerol in the skin, thereby inhibiting the growth of *Propionibacterium acnes*, which is related to the formation of acne (Iwase et al., 2010). The phenomenon of growth interference between *Propionibacterium acnes* and *Staphylococcus epidermidis* through fermentation has been used to develop probiotic products for acne and other skin diseases. Cho BO et al. found that soybean fermented with *Bacillus amyloliquefaciens* SCGB1 could be considered as a promising functional food for managing clinical, histological and immunological spectra associated with Atopic Dermatitis (Cho et al., 2019).In this study, cosmetics containing complex polysaccharides may reduce the potential acne-causing *Propionibacterium* by adding beneficial *Staphylococcus*. In addition, *Bifidobacterium* is an important and beneficial intestinal microbe of humans and animals. Probiotics and/or prebiotics, such as *Bifidobacterium breve* strain Yakult and/or GOS, are expected to help maintain a healthy skin by decreasing phenols production by gut microbiota (Miyazaki et al., 2014). Some bifidobacterial species and strains have been reported to improve AD *via* modulating immune-microbe interactions in patients. Fang et al. suggested that AD symptoms may be alleviated by modifying I3C, an AHR agonist produced by *Bifidobacterium longum* CCFM1029, which triggers communication between the gut and the skin (Fang et al., 2022). Sukyung et al. found that oral administration of *Bifidobacterium longum* and GOS can improve DNCB-induced skin barrier dysfunction and atopic dermatitis like skin (Kim et al., 2022). The *bifidobacterium* mentioned in these studies acts through the gut-skin axis, however, the abundance of *bifidobacterium* in the skin, as well as its role, has rarely been reported. Interestingly, our study found that cosmetics containing complex polysaccharides can significantly reduce the content of bifidobacteria in cheek skin bacterial microbiota, although the mechanism may require further research and discussion. Butler et al. have pointed out that bacteria, mainly *Lactobacillus*, can also be active ingredients in cosmetics (Butler et al., 2020). Their antagonistic activity against pathogens may result from the competition, synthesis, and secretion of various antimicrobial substances or the blocking of their adhesion to skin cells. They used live *Lactobacillus reuteri* DSM 17938 as the active ingredient of a new type of topical cosmetics ointment, which may become a standard topical product for the treatment of atopic dermatitis or other skin-related diseases. Another study showed that base cream including heat-killed *Lactobacillus plantarum* GMNL6 could regulate human skin health by improving skin microbiota (Tsai et al., 2021). In our study, compared with the baseline skin bacterial microbiota, after 28 days of using complex polysaccharide cosmetics, the relative abundance of Lactobacillus in the cheek and forehead was reduced from 1.1% to 0.3% and 1.5% to 0.4%, respectively. Although Lactobacillus may have a therapeutic effect on some skin diseases, it only targets certain strains. Therefore, the reduction of *Lactobacillus* cannot be arbitrarily defined as harmful but should be specific to the level of species or even strains. We did not perform abundance determination of specific strains, which may be a shortcoming of this research, and needs to be further investigated in future studies.

## Conclusion

In conclusion, the main genera of the forehead skin bacterial microbiota were *Cutibacterium*, *Acinetobacter*, *Enterococcus*, *Ralstonia*, and *Staphylococcus*, while those of the cheek skin bacterial microbiota were *Staphylococcus*, *Ralstonia*, *Propionibacterium*, *Acinetobacter*, and *Bifidobacterium*. Furthermore, cosmetics containing complex polysaccharides can help to maintain stable conditions of the skin by changing the amount and diversity of the skin bacterial microbiota.

## Data availability statement

The datasets presented in this study can be found in online repositories. The names of the repository/repositories and accession number(s) can be found at: https://www.ncbi.nlm.nih.gov/, PRJNA950215.

## Ethics statement

The studies involving human participants were reviewed and approved by the Ethics Committee in Jiangnan University, China (SYXK 2012-0002). The patients/participants provided their written informed consent to participate in this study.

## Author contributions

Conceptualization, SC, KY and JZ; methodology, MP and XT; software, MP and BM; investigation, SC and MP; resources, GL, and JZ; writing—original draft preparation, SC and MP; writing—review and editing, BM and JZ; supervision, JZ and KY. All authors contributed to the article and approved the submitted version.
