# Supplementary material for: Metagenomic insights into the effects of cosmetics containing complex polysaccharides on the composition of skin microbiota in females
Source: Front Cell Infect Microbiol. 2023 Aug 1;13:1210724. doi: 10.3389/fcimb.2023.1210724 (PMC10428012; doi:10.3389/fcimb.2023.1210724)
Supplement: Supplementary file 1 [file DataSheet_1.docx]

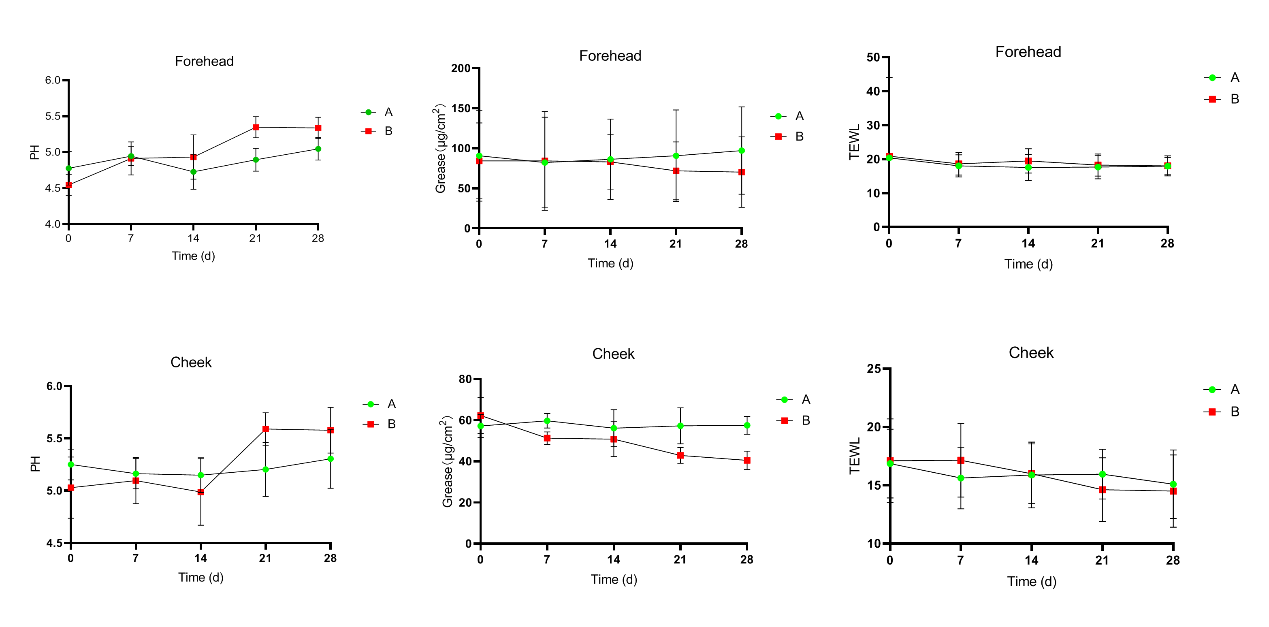


Figure S1. The facial skin physiological indicators of volunteers in Group A and Group B


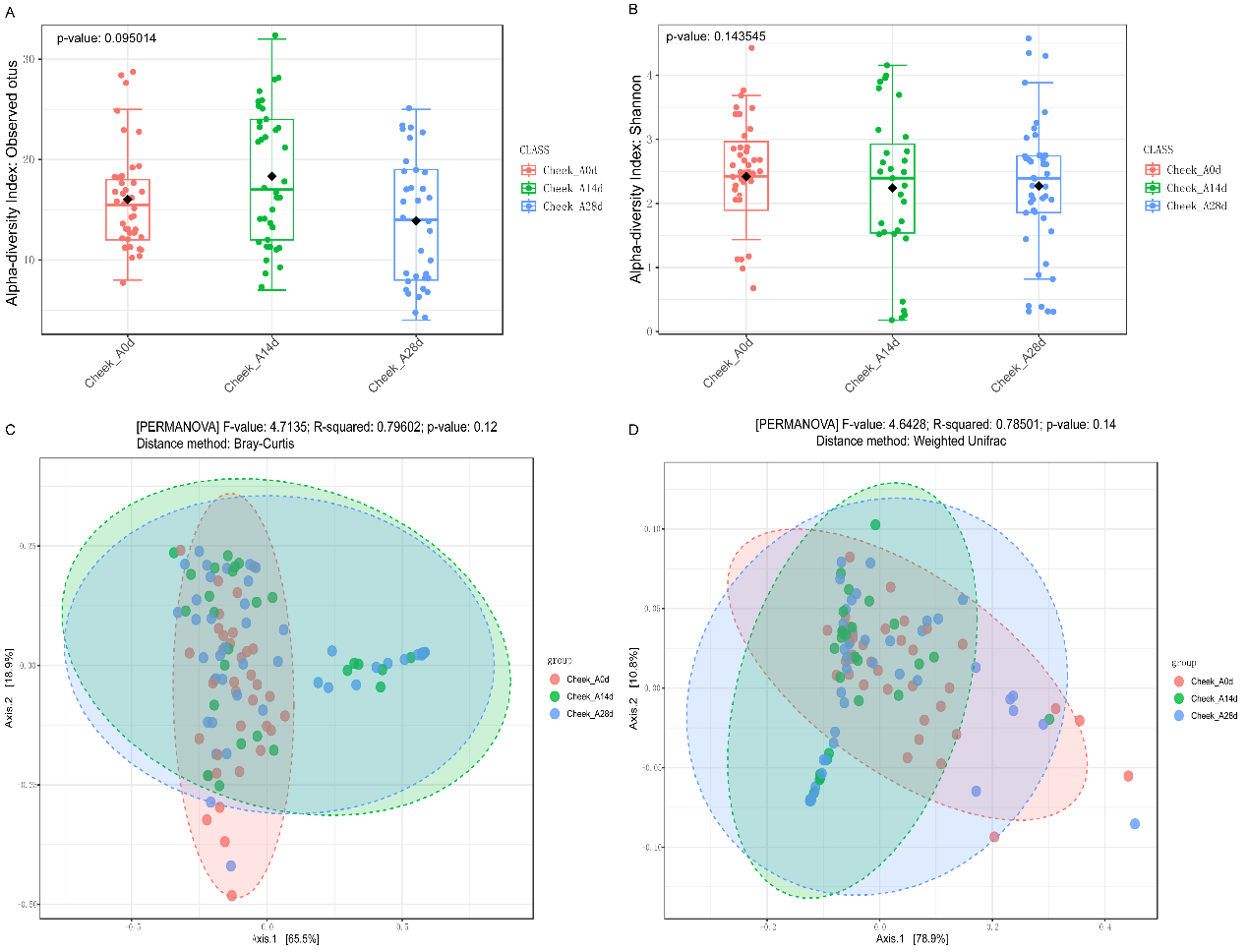


Figure S2. The aphla and beta diversity of cheek skin in Group A


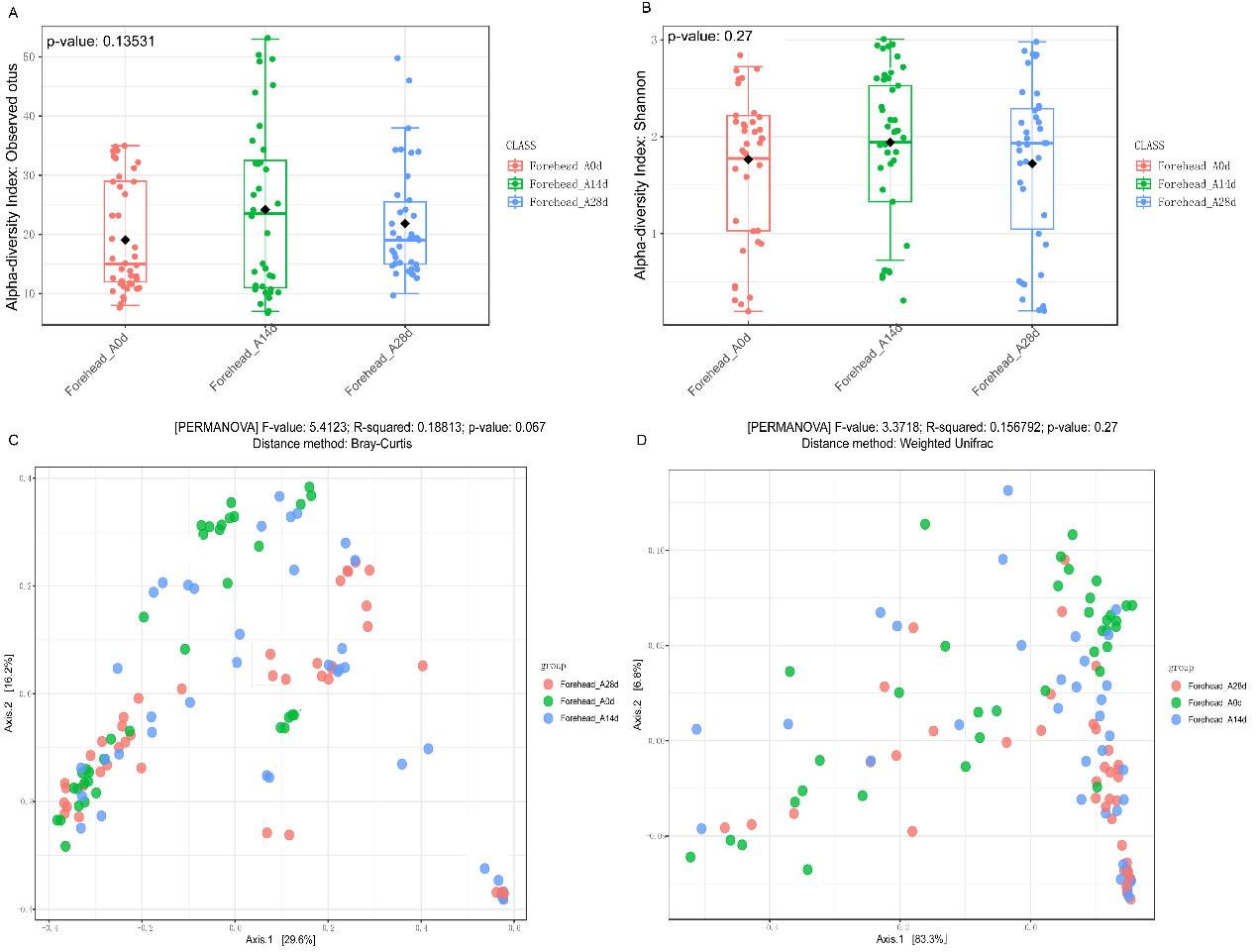


Figure S3. The aphla and beta diversity of forehead skin in Group A

Table S1 The age of volunteers in Group A and Group B

| **ID** | **Age** | **Group** | **ID** | **Age** | **Group** |
| --- | --- | --- | --- | --- | --- |
| 1 | 20 | A | 38 | 41 | B |
| 2 | 28 | A | 39 | 23 | B |
| 3 | 25 | A | 40 | 48 | B |
| 4 | 26 | A | 41 | 20 | B |
| 5 | 33 | A | 42 | 50 | B |
| 6 | 27 | A | 43 | 22 | B |
| 7 | 30 | A | 44 | 20 | B |
| 8 | 50 | A | 45 | 21 | B |
| 9 | 45 | A | 46 | 20 | B |
| 10 | 54 | A | 47 | 20 | B |
| 11 | 23 | A | 48 | 42 | B |
| 12 | 22 | A | 49 | 51 | B |
| 13 | 26 | A | 50 | 20 | B |
| 14 | 32 | A | 51 | 27 | B |
| 15 | 25 | A | 52 | 20 | B |
| 16 | 27 | A | 53 | 45 | B |
| 17 | 26 | A | 54 | 39 | B |
| 18 | 26 | A | 55 | 22 | B |
| 19 | 25 | A | 56 | 33 | B |
| 20 | 24 | A | 57 | 26 | B |
| 21 | 23 | A | 58 | 24 | B |
| 22 | 26 | A | 59 | 21 | B |
| 23 | 29 | A | 60 | 24 | B |
| 24 | 55 | A | 61 | 26 | B |
| 25 | 21 | A | 62 | 45 | B |
| 26 | 37 | A | 63 | 21 | B |
| 27 | 22 | A | 64 | 20 | B |
| 28 | 26 | A | 65 | 46 | B |
| 29 | 26 | A | 66 | 25 | B |
| 30 | 38 | A | 67 | 32 | B |
| 31 | 40 | A | 68 | 25 | B |
| 32 | 25 | A | 69 | 29 | B |
| 33 | 29 | A | 70 | 30 | B |
| 34 | 35 | A | 71 | 24 | B |
| 35 | 37 | A | 72 | 20 | B |
| 36 | 23 | A | 73 | 51 | B |
| 37 | 20 | A | 74 | 23 | B |
